# Endothelial-Rac1 Is Not Required for Tumor Angiogenesis unless αvβ3-Integrin Is Absent

**DOI:** 10.1371/journal.pone.0009766

**Published:** 2010-03-22

**Authors:** Gabriela D'Amico, Stephen D. Robinson, Mitchel Germain, Louise E. Reynolds, Gareth J. Thomas, George Elia, Garry Saunders, Marcus Fruttiger, Victor Tybulewicz, Georgia Mavria, Kairbaan M. Hodivala-Dilke

**Affiliations:** 1 Adhesion and Angiogenesis Laboratory, Institute of Cancer, Bart's and The London, Queen Mary's School of Medicine and Dentistry, London, United Kingdom; 2 Centre for Tumour Biology, Institute of Cancer, Bart's and The London, Queen Mary's School of Medicine and Dentistry, London, United Kingdom; 3 Institute of Ophthalmology, University College London, London, United Kingdom; 4 Division of Immune Cell Biology, National Institute for Medical Research, London, United Kingdom; 5 Cancer Research UK Centre for Cell and Molecular Biology, Institute of Cancer Research, London, United Kingdom; Leiden University, Netherlands

## Abstract

Endothelial cell migration is an essential aspect of tumor angiogenesis. Rac1 activity is needed for cell migration *in vitro* implying a requirement for this molecule in angiogenesis *in vivo*. However, a precise role for Rac1 in tumor angiogenesis has never been addressed. Here we show that depletion of endothelial Rac1 expression in adult mice, unexpectedly, has no effect on tumor growth or tumor angiogenesis. In addition, repression of Rac1 expression does not inhibit VEGF-mediated angiogenesis *in vivo* or *ex vivo*, nor does it affect chemotactic migratory responses to VEGF in 3-dimensions. In contrast, the requirement for Rac1 in tumor growth and angiogenesis becomes important when endothelial **β3**-integrin levels are reduced or absent: the enhanced tumor growth, tumor angiogenesis and VEGF-mediated responses in **β3**-null mice are all Rac1-dependent. These data indicate that in the presence of αv**β3**-integrin Rac1 is not required for tumor angiogenesis.

## Introduction

Tumor growth and metastasis are both dependent on the development of a tumor vasculature in a process termed angiogenesis. This process involves changes in the migration and proliferation of endothelial cells to form capillaries which invade into the growing tumor mass to supply it with nutrients and oxygen [1]. The list of molecular co-ordinators of angiogenesis is vast and includes some members of the family of extracellular adhesion molecules, integrins, and several growth factors and their receptors such as vascular endothelial growth factor (VEGF) and VEGF-receptor2 (VEGFR2) [2]. Blood vascular endothelial cells express several integrins including αv**β3**, a predominant vitronectin receptor. Inhibitors of αv**β3** have been developed as anti-angiogenic agents for the treatment of cancer [2]. Indeed αv**β3**- and VEGF-inhibitors, used in combination with chemotherapy, are both in Phase III clinical trials, the former for the treatment of glioblastoma and the latter especially for colorectal cancer [3], [4], [5], [6], [7]. However, neither anti-integrin nor anti-VEGF treatments are effective enough at blocking angiogenesis to completely halt cancer progression and, at best, can extend mean patient survival by a few months [8], [9], [10], [11]. In contrast to the inhibition studies, global genetic ablation of **β3**-integrin results in viable, fertile animals which display enhanced pathological angiogenesis due in part to the up-regulation of VEGFR2 [12], whereas in a transgenic model, where the cytoplasmic tail of **β3**-integrin is mutated, pathological angiogenesis is impaired [13]. Taken together these data demonstrate that the molecular mechanisms by which these molecules regulate angiogenesis are complex and in need of further investigation.

There are several examples where the levels of **β**3-integrin have been reported to be undetectable in developing blood vessels. Apart from the lack of **β**3-integrin in Glanzmann thromabastenic patients, there are several physiological examples where **β**3 levels are low or undetectable: (1) during embryonic development, **β**3-integrin expression is barely detectable until mouse embryonic day E15.5 onwards, before which, most developmental angiogenesis is already well established [14]; (2) **β**3-integrin expression is not observed in the vasculature of healing liver [15]; (3) the vasculature of angiosarcomas has undetectable levels of **β**3-integrin [16]; (4) the vasculature of lung metastases originated from colorectal cancer also lack **β**3-integrin [17], [18]; (5) the blood vessels in neuroendocrine tumors also show no significant change in **β**3-integrin levels between normal and tumor tissue [19]; (6) in Lewis cell lung carcinomas, **β**3-integrin expression is absent until the tumors reach a relatively large size of 500 mm^3^ or more suggesting that the initial stages of angiogenesis lack **β**3-integrin [15]. Taken together, these types of data suggest that screening for **β**3-integrin levels, will be important in better detecting which tumor types will be responsive to **β**3-specific anti-angiogenic therapy. Indeed the lack of an anti-tumor effect of many of the anti-αv**β**3-inhibitors, except for in glioblastma, suggest that perhaps **β**3-integrin in the vasculature of some tumors is not significantly high or functional. Thus it is of importance to understand the molecular basis of **β**3-integrin low/deficient angiogenesis.

Downstream signalling pathways of both integrins and growth factor receptors involve RhoA, Cdc42 and Rac1, all members of the family of Rho GTPases. RhoA, Cdc42 and Rac1 can be activated when GTP-bound, and inactivated, when GDP-bound. The activation of RhoA has been reported to be involved in the development of actin stress fibres, Cdc42 in filopodia formation and Rac1 in membrane-ruffling [20]. Rac1 has been shown to be required for the migration of cells, including endothelial cells, and also it appears to play a role in vascular tube formation and lumen formation, at least *in vitro* [21], [22]. Its involvement in the function of endothelial cells has also been illustrated by the activation of Rac1 under flow and shear stress [23]. Recently, some studies have suggested a role for Rac1 in endothelial cells *in vivo*. For example, haploinsufficiency of Rac1 in Tie2-Cre mice results in the poor regulation of blood pressure, vasodilation and impaired hind limb ischemia-induced neovascularization, suggesting that a 50% loss of Rac1 in Tie2-Cre mice is sufficient to affect this process [24]. In addition, Rac1-deletion in Tie2-Cre mice has implied a role for Rac1 in developmental vasculogenesis, due, possibly, to its deletion both in Tie2-positive endothelial cells and in approximately 80% of hematopoetic cells which are also Tie2-positive [25]. However, since these embryos die at E9.5, before developmental angiogenesis can proceed fully, the precise effect of endothelial-Rac1-deficiency in tumor angiogenesis *in vivo* is unknown.

Here we show that, surprisingly, the inducible-deletion of Rac1 in adult endothelial cells does not affect tumor angiogenesis or VEGF-mediated angiogenesis in adult mice. However, in the absence of **β3**-integrin, the dependency of these processes on Rac1 becomes apparent. Our data indicate that Rac1, in adult endothelial cells, is not normally required for the migration and capillary formation necessary in adult neovascularization in vivo and that the role for this Rho GTPase *in vivo* may well be different to its functions *in vitro*.

## Results

### Tumor growth and tumor angiogenesis in β3-null, but not wild-type mice, are dependent on adult endothelial Rac1 expression

**Figure 1:**
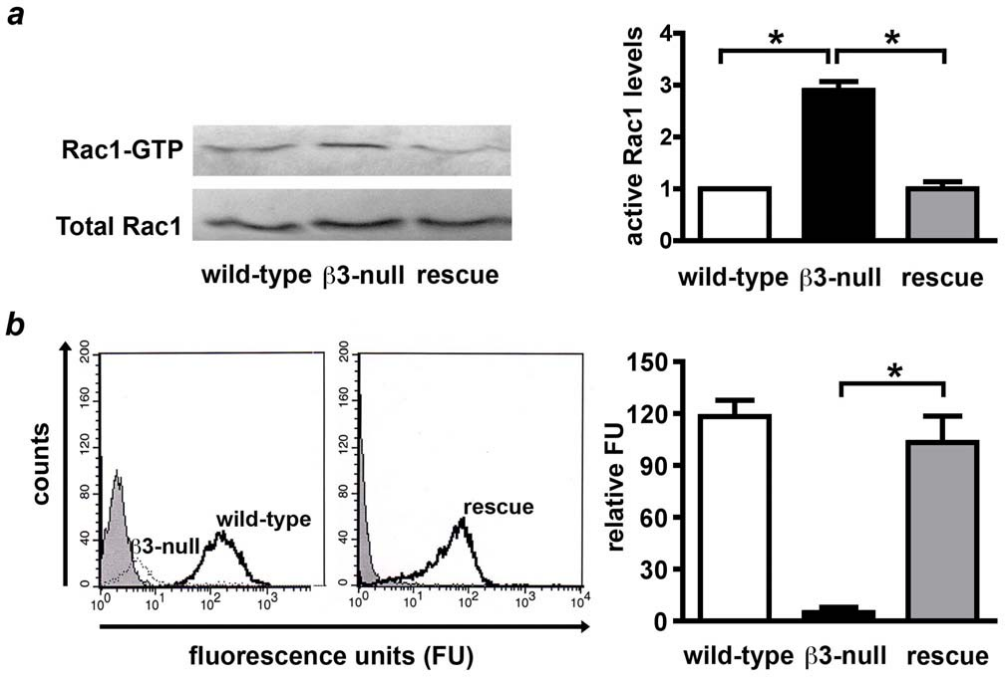
Increased active levels of Rac1 in β3-integrin-deficient endothelial cells. **A.** Active levels of Rac1 were examined by GST-PAK pull-downs. Western blot analysis of active Rac1 bound to GST-PAK (Rac1-GTP) and total Rac1 from wild-type, β3-integrin null, and β3-null primary lung endothelial cells transduced with human β3-integrin (rescue). Immunoblots were quantified by densitometry, and the levels of GTP-bound Rac1 normalised to total Rac1 levels. Active levels of Rac1 were increased approximately 3 fold in β3-null endothelial cells when compared with wild-type controls (*P<0.01), however, total levels of Rac1 were expressed equally in both genotypes. Furthermore, active levels of Rac1 were reduced to wild-type levels in rescue cells (*P<0.01). Results shown are the means + s.e.m of 3-4 independent experiments. **B.** Flow-cytometric analysis shows that surface levels of β3-integrin were not detectable in β3-null (dashed line) cells when compared with wild-types (bold line) endothelial cells (left panel). Rescue cells expressed β3-integrin (bold line, right panel). Grey peaks represent isotope IgG controls. Bar graph shows means + s.e.m of relative surface β3-integrin expression in wild-type (white), β3-null (black) and rescue (grey) endothelial cells compared with negative control (*P<0.001, N = 3 independent experiments).

Endothelial cell migration is a critical feature of tumor angiogenesis and is thought to involve both changes in integrin expression and the activity of Rho GTPases such as Rac1 [26]. However, a precise role for Rac1 in tumor angiogenesis has never been addressed. We provide evidence that Rac1 activity (Rac1-GTP) is elevated in **β3**-integrin knockout endothelial cells without affecting total Rac1 levels, and that transduction of human **β3**-integrin into **β3**-null endothelial cells is sufficient to reduce Rac1-GTP levels back to wild-type levels (**Figure 1A, B**). Given the enhanced pathological angiogenic responses in **β3**-null mice [12] we asked whether endothelial Rac1 was differentially required in tumor angiogenesis in adult wild-type or **β3**-null mice.

To investigate whether Rac1-deficiency in endothelial cells affected tumor growth and tumor-associated angiogenesis in adult mice, a lentiviral vector for conditional, Cre-lox regulated, stable RNA interference (RNAi) was employed (pSico-Rac1). This vector allows tissue-specific activation of Rac1 short hairpin RNA (shRNA) in Cre-expressing cells *in vivo*. As a positive control (for inhibiting angiogenesis), pSico-Flk-1 was employed for the depletion of Flk-1 (VEGFR2). As a negative control pSico-Con, targeting firefly luciferase was also used.

**Figure 2:**
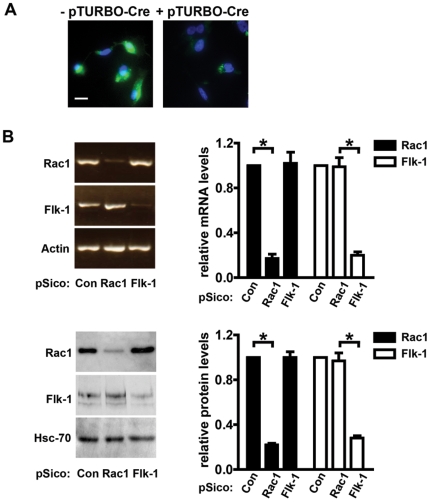
Cre-regulated depletion of Rac1. **A.** Wild-type primary endothelial cells were infected with pSico-Rac1 lentivirus. High-efficiency transduction was achieved as indicated by uniform GFP expression in infected cells (left panel). Cells sorted for GFP positivity and transfected with a Cre-recombinase expression plasmid (pTURBO-Cre) showed a significant loss of GFP (right panel). Scale bar: 10 µm. **B.** One week after pTURBO-Cre transfection, levels of mRNA, detected by semi-quantitative RT-PCR (upper panels), and protein, detected by Western blotting (lower panels), showed successful Rac1- and Flk-1-depletion in pSico-Rac1 and pSico-Flk-1 infected cells, respectively. Αctin RT-PCR and Western blotting for Hsc-70 were carried out to ensure equal RNA and protein loading, respectively. Bar graphs represent densitometric readouts of mRNA of relative Actin or Hsc-70 protein levels, respectively. *P<0.01. N = 3 independent experiments.

The efficiency of the system was first tested *in vitro*. The pSico plasmid contains a GFP-reporter that is excised after Cre-mediated recombination and shRNA expression. Wild-type primary endothelial cells infected with pSico-Rac1 lentiviruses were analyzed for GFP-expression and showed high-efficiency transduction. GFP-positive cells were then isolated by FACS and transfected with a Cre expression-plasmid (pTURBO-Cre). Near complete recombination, with a subsequent loss of GFP expression, was observed for all transduced cells (P<0.01, **Figure 2A**). Moreover, a significant reduction of both Rac1 and Flk-1 mRNA and protein levels was achieved in pSico-Rac1 or pSico-Flk-1 transduced cells, respectively. As expected, no significant changes in either mRNA or protein levels of Rac1 or Flk-1 were observed in pSico-Con infected cells (**Figure 2B**).

**Figure 3:**
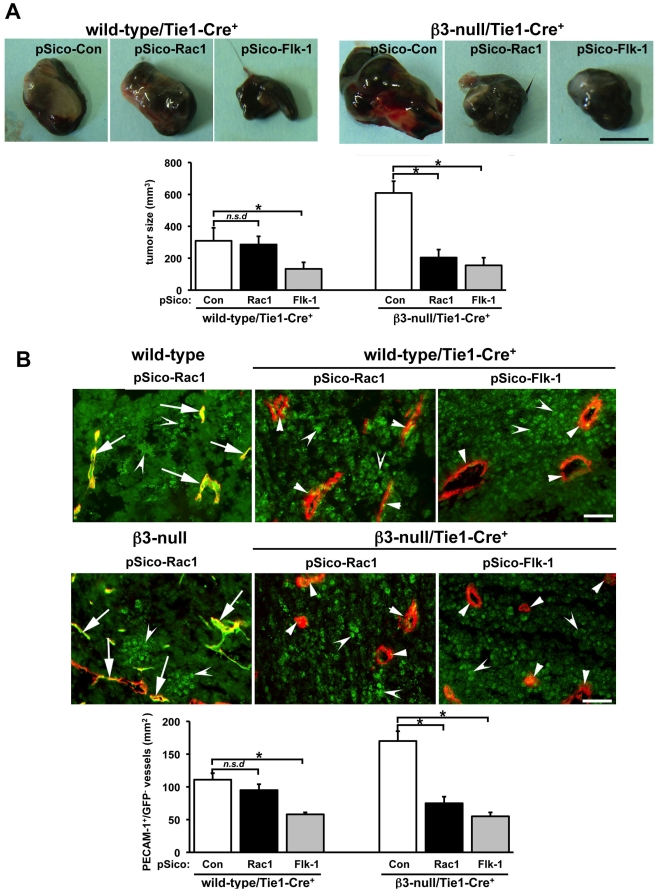
Endothelial-specific Rac1-depletion does not impair tumor growth wild-type mice but does in β3-null mice. **A.** Murine B16F0 cells (10^6^) were injected subcutaneously into the flanks of wild-type/Tie1-Cre^+^ or β3-null/Tie1-Cre^+^ mice. Lentiviral vector suspensions (10^6^ i.u/ml) of pSico-Con, pSico-Rac1 and pSico-Flk-1 were injected intratumorally on days 5 and 10 after tumor cell injection. Representative macroscopic appearance of 14-day-old B16F0 pSico-Con-, pSico-Rac1- and pSico-Flk-1- treated melanomas in both genotypes. Scale bar: 5 mm. Bar graph shows mean tumor volume per mm^3^ (+ s.e.m.). Tumor size was reduced significantly in pSico-Flk-1-treated mice of both genotypes (*P<0.05) and in pSico-Rac1 treated β3-null/Tie1-Cre^+^ but not in pSico-Rac1-treated wild-type/Tie1-Cre^+^ mice (*n.s.d*, no significant differences). N = 4–6 animals per condition. **B.** Representative merged images of PECAM-1 (red) and GFP (green) -immunostained sections from pSico-Rac1-treated B16F0 tumors grown in wild-type, wild-type/Tie1-Cre^+^, β3-null and β3-null/Tie1-Cre^+^ mice. PECAM-1-positive staining identified endothelium. GFP-positive staining was observed in tumor cells (concave arrowheads) and in PECAM+ endothelium (arrows) of blood vessels in B16F0 tumors from wild-type and β3-null control mice, indicating successful pSico-Rac1 lentivirus infection *in vivo*. Loss of GFP detection in most PECAM-1-positive microvessels (small arrowheads), but not in B16F0 tumor cells, was observed in pSico-treated tumors grown in wild-type/Tie1-Cre^+^ and β3-null/Tie1-Cre^+^ mice, indicating successful endothelial-specific Cre recombination *in vivo*. Scale bar: 10 µm. Bar graph shows mean numbers of PECAM-1^+^/GFP^−^ vessels per unit area of tumor section per mm^2^ (+ s.e.m). Blood vessel density was reduced significantly in pSico-Flk-1-treated mice of both wild-type/Tie1-Cre^+^ and β3-null/Tie1-Cre^+^ mice (*P<0.05) and in pSico-Rac1-treated β3-null/Tie1-Cre^+^ mice but not pSico-Rac1-treated wild-type/Tie1-Cre^+^ mice (*n.s.d*, no significant differences). N = 4 animals per condition.

To study the effect of pSico-Rac1 on tumor growth, wild-type/Tie1-Cre^+^ and **β3**-null/Tie1-Cre^+^ mice were injected subcutaneously with murine B16F0 melanoma cells and pSico-Con, pSico-Rac1 or pSico-Flk-1 lentiviral suspensions injected intratumorally on days 5 and 10 after tumor cell inoculation. In line with our previous findings [12], [27], tumor size in pSico-Con treated **β3**-null/Tie1-Cre^+^ mice was greater than similarly treated wild-type/Tie1-Cre^+^ mice. Results also showed that although pSico-Flk-1 treatment reduced significantly B16F0 melanoma size (P<0.05) in both genotypes, when compared with pSico-Con controls, pSico-Rac1-treatment did not affect tumor size in wild-type/Tie1-Cre^+^ mice, but significantly reduced tumor size in **β3**-null/Tie1-Cre^+^ mice (**Figure 3A**).

To determine the efficacy of the lentiviruses *in vivo*, double-immunostaining for PECAM-1 and GFP was performed in sections of tumors grown in wild-type/Tie1-Cre^−^ or wild-type/Tie1-Cre^+^ mice treated with either pSico-Rac1 or pSico-Flk-1 lentiviruses. Immunostaining for PECAM-1 identified blood vessels under both conditions. In wild-type/Tie1-Cre^−^ mice, GFP expression was detected in both the tumor cells and the majority of endothelial cells lining blood vessels, indicating a high efficiency of lentiviral infection *in vivo*. In contrast, analysis of tumors grown in wild-type/Tie1-Cre^+^ mice showed a loss of GFP expression in endothelial cells but not tumor cells, indicating that endothelial-specific Cre-recombination was efficient (**Figure 3B**). Taken together, these data indicate that, while no biological effect of Rac-1 knockdown was observed in wild-type/Tie1-Cre^+^, the lentivirus treatment efficiently reduced expression of Rac1 in tumor blood vessels from these animals.

To examine the effect of endothelial Rac1-depletion on tumor angiogenesis, blood vessel density was quantified by counting the numbers of PECAM-1^+^/GFP^−^ blood vessels per unit area across entire midline tumor sections from size-matched tumors of wild-type/Tie1-Cre^+^ and **β3**-null/Tie1-Cre^+^ mice treated with either pSico-Con, pSico-Rac1 or pSico-Flk-1 lentiviruses. Once again, concurrent with our previous work [12], [27], blood vessel density in pSico-Con treated **β3**-null/Tie1-Cre^+^ mice was significantly greater than in pSico-Con treated wild-type/Tie1-Cre^+^ mice. However, although pSico-Flk-1 treatment inhibited significantly tumor angiogenesis in both wild-type/Tie1-Cre^+^ and **β3**-null/Tie1-Cre^+^ mice (P<0.05), pSico-Rac1-treatment did not affect tumor angiogenesis in wild-type/Tie1-Cre^+^ but did inhibit tumor angiogenesis significantly in **β3**-null/Tie1-Cre^+^ mice (**Figure 3B**).

These results indicate that Rac1-depletion in tumor endothelial cells does not affect tumor growth or tumor angiogenesis in wild-type/Tie1-Cre^+^ mice but does inhibit both these processes in **β3**-null/Tie1-Cre^+^ mice.

**Figure 4:**
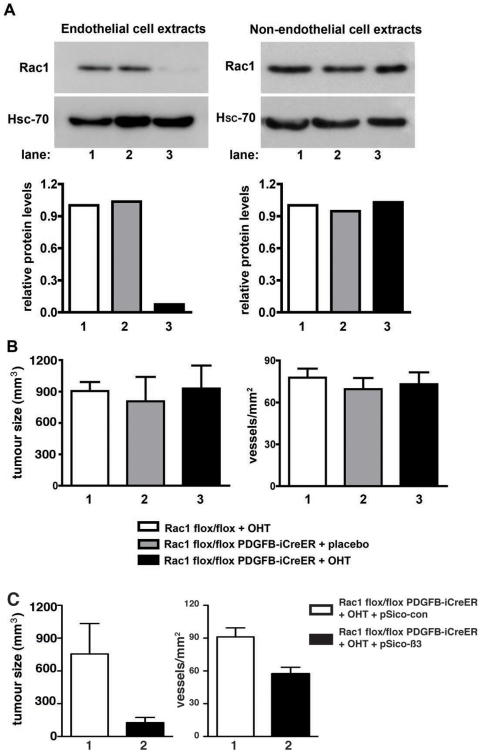
Genetic ablation of Rac1 in endothelial cells does not impair tumor growth or tumor angiogenesis. **A.** Western blot analysis of Rac1 flox/flox PDGFB-iCreER OHT-treated primary endothelial cell extracts showed that Rac1 was barely detectable (lane 3) when compared with Rac1 flox/flox OHT-treated (lane 1) or Rac1 flox/flox PDGFB-iCreER vehicle treated (lane 2) endothelial cell extracts (left panel). Western blots of extracts from non-endothelial cells, treated as described above, showed no differences in Rac1 expression (right panel). Hsc-70 provided loading controls. Bar graphs represent densitometric values relative to Hsc-70. N = 2 independent experiments. **B.** B16F0 tumor volume and angiogenesis (blood vessel density) from tumors grown in OHT-treated Rac1 flox/flox (1, white), or placebo- (2, grey) or OHT- (3, black) treated Rac1 flox/flox PDGFB-iCreER mice. Left bar graph shows mean tumor volume in mm^3^ (+ s.e.m) for 10-day-old tumors. No significant differences in tumor size were observed between groups. N = 6–7 animals per group. Right bar graph shows mean number of PECAM-1 positive vessels per tumor area per mm^2^ (+ s.e.m.) for 10-day-old tumors. No significant differences in microvessel density were observed between groups. N = 4 animals per group. **C.** B16F0 tumor volume and angiogenesis (blood vessel density) from tumors grown in OHT-treated Rac1 flox/flox PDGFB-iCreER animals. Tumors were injected at days 5 and 10 (after initial inoculation) with pSico-Con (1, white) or pSico- β3 (2, black) and harvested at day 14. Left bar graph shows mean tumor volume in mm^3^ (+ s.e.m). *P*<0.05. N = 5 animals per group. Right bar graph shows mean number of endomucin positive vessels per tumor area per mm^2^ (+ s.e.m.). *P*<0.001. N = 5 animals per group.

Further confirmation of the role of endothelial Rac1 in tumor growth and angiogenesis was accomplished by using a genetic ablation approach that allows conditional Rac1-deletion in an inducible-manner. Rac1-floxed mice [28] were crossed with PDGFB-iCreER transgenic mice (23) (where Cre expression, in adult animals, is restricted primarily to angiogenic/proliferating endothelial cells [29]) to generate tamoxifen-inducible endothelial-specific Rac1-deficient mice (Rac1 flox/flox PDGFB-iCreER). To verify that tamoxifen treatment of Rac1 flox/flox PDGFB-iCreER endothelial cells induced Rac1-deletion, endothelial cells and non-endothelial cells were isolated from Rac1 flox/flox PDGFB-iCreER mice and treated with tamoxifen (OHT) or ethanol (vehicle) in culture. Rac1 protein levels were barely detected in Rac1 flox/flox PDGFB-iCreER tamoxifen-treated endothelial cells when compared with vehicle-alone treated Rac1 flox/flox PDGFB-iCreER or tamoxifen-treated Rac1 flox/flox (PDGFB-iCreER negative) controls. Importantly, tamoxifen treatment of non-endothelial cells isolated from Rac1 flox/flox PDGFB-iCreER mice had no effect on Rac1 expression (**Figure 4A**). Rac1 flox/flox PDGFB-iCreER mice were injected subcutaneously with B16F0 cells and, one day later, treated with tamoxifen (OHT) or placebo administered by slow-release pellets implanted subcutaneously to induce Cre-activity in PDGFB-positive cells. Deletion of Rac1 in the vascular endothelium did not affect tumor size or tumor angiogenesis when compared with controls (**Figure 4B**).

As yet an additional test of the roles played by Rac1 and β3-integrin in angiogenesis *in vivo*, Rac1 flox/flox PDGFB-iCreER animals received tamoxifen pellet implants. These animals then were injected subcutaneously with B16F0 cells and subsequently received intra-tumoral injections of pSico-Con (as described above) or a pSico lentivirus directed against β3-integrin (pSico-β3). Tumors treated with pSico-Con grew to an expected size of approximately 800 mm^3^. pSico-β3 treated tumors, however, were significantly smaller (*P*<0.05, **Figure 4C**). Blood vessel counts of endomucin stained tumor sections showed a significant reduction in angiogenesis after treatment with pSico−β3 (*P*<0.001, **Figure 4C**). Taken together, data from Rac1 flox/flox PDGFB-iCreER animals provided a second line of evidence that tumor growth and angiogenesis *in vivo* does not normally depend on Rac1.

**Figure 5:**
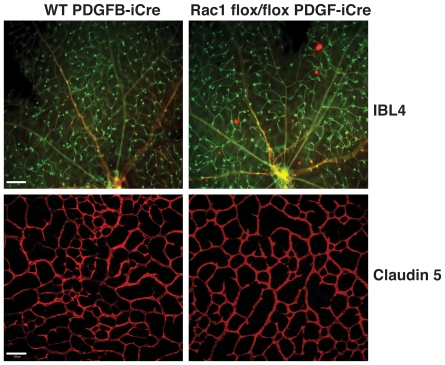
Postanatal retinal vascularization is not affected by endothelial Rac1 deletion. Wild-type PDGFB-iCreER and PDGFB-iCreER; Rac1 flox/flox newborn pups were treated daily with tamoxifen and their retinas were examined at P12. The vasculature was visualised by Interleukin β4 staining (ILB4 - green) and Claudin 5 (red). No obvious differences in vascularization were observed between genotypes. Scale bar: 10 µm upper micrographs; 100 µm lower micrographs.

Already published studies suggest that Rac1 plays a role in vascular tube formation, at least *in vitro* [21], [22]. As such, the data describe here were somewhat surprising. To further test the effect of Rac1 deletion in another neovascularization assay, the influence of Rac1 deletion was analyzed in the post-natal developing retina [29]. Wild-type PDGFB-iCreER and Rac1 flox/flox PDGFB-iCreER newborn pups were treated daily with tamoxifen by oral gavage and their retinas were examined at P12. No significant differences in central primary plexus, or peripheral primary plexus branch points were observed between the two genotypes (**Figure 5**). Therefore, deficiency of Rac1 in endothelial cells is not sufficient to alter neovasculatization *in vivo*, at least when β3-integrin is present.

Using multiple *in vivo* approaches, we demonstrated that endothelial Rac1 is not essential for tumor growth or tumor angiogenesis in adult wild-type mice, but that these processes become Rac1-dependent in the absence of **β3**-integrin.

### Rac1 is not required for VEGF-mediated angiogenesis in wild-type mice but is in β3-null mice

**Figure 6:**
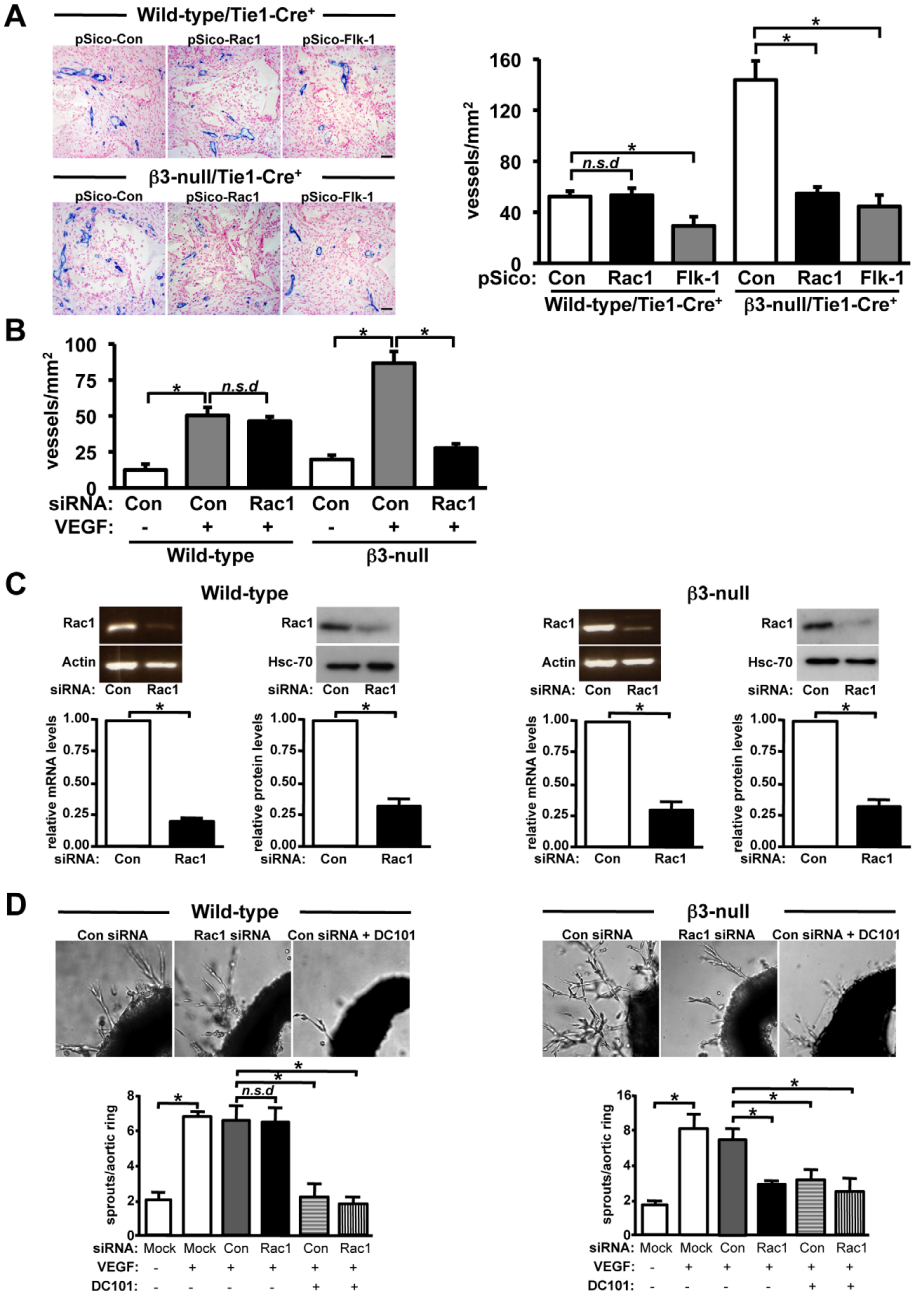
VEGF-mediated angiogenesis is dependent on endothelial-Rac1 expression in β3-null mice but not wild-type mice. **A.** Representative images of 14-day-old VEGF-impregnated sponges in wild-type/Tie1-Cre^+^ and β3-null/Tie1-Cre^+^ mice after treatment with pSico-Con, pSico-Rac1 and pSico-Flk-1. Endomucin-positive staining (blue) identified microvessels. Scale bar: 50 µm. Bar graph represents mean number of microvessels per area of sponge (+ s.e.m.). Blood vessel density was reduced significantly in pSico-Flk-1-treated mice of both genotypes (*P<0.05), and in pSico-Rac1-treated β3-null/Tie1-Cre^+^ mice, but not pSico-Rac1-treated wild-type/Tie1-Cre^+^ mice (*n.s.d*, no significant differences). N = 6 sponges per group. **B.** Subcutaneous sponges implanted into wild-type mice were injected with either PBS or VEGF in the presence (Rac1 siRNA) or absence (Con siRNA) of Rac1-specific siRNA. Bar graph shows mean number of laminin-positive vessels per area of sponge per mm^2^ (+ s.e.m). Blood vessel density was increased significantly in VEGF alone-treated (*P<0.01) when compared with PBS controls. Rac1 siRNA-treatment had no significant effect (*n.s.d*, no significant differences). N = 6–10 sponges per group. **C.** Semi-quantitative RT-PCR (left) and Western blot (right) analyzes from wild-type and β3-null aortic explants transfected with either Con or Rac1 specific-siRNA. Rac1 siRNA reduced significantly the expression of both Rac1 mRNA (*P<0.01) and protein (*P<0.001) levels. RT-PCR for Αctin and Western blotting for Hsc-70 provided mRNA and protein loading controls, respectively. Bar graphs represent mean Rac1 mRNA and Rac1 protein levels (+ s.e.m.). N = 3 independent experiments. **D.** *Ex vivo* mouse aortic ring assays. Representative high-power light micrographs of VEGF-mediated microvessel sprouting from wild-type and β3-null mouse aortic rings treated with Con or Rac1-specific siRNA and Con siRNA plus DC101. Bar graphs represent quantification of microvessel numbers from 5-day-old VEGF-stimulated aortic ring cultures transfected with Mock, Con or Rac1 siRNA in the presence or absence of DC101. Sprouting angiogenesis was reduced significantly after DC101 treatment of aortas and in Rac1-specific siRNA transfected β3-null, but not wild-type, aortas. Bar graph represents mean number microvessel sprouts/aortic ring (+ s.e.m.).*P<0.01; *n.s.d*, no significant differences. N = 3-5 independent experiments.

Given that VEGF is a key positive regulator of pathological angiogenesis and that VEGF/VEGFR2 signalling is known to activate Rac1 *in vitro*, we tested the effect of endothelial-specific Rac1-depletion on VEGF-mediated neovascularization *in vivo*. Synthetic sponges were implanted subcutaneously into wild-type/Tie1-Cre^+^ and **β3**-null/Tie1-Cre^+^ mice and injected *in situ* with 10 ng/ml VEGF-A164 in combination with either pSico-Con, pSico-Rac1 or pSico-Flk-1 lentiviral suspensions. After 15-days, angiogenic responses were assayed histologically. Results showed that, as expected, VEGF-mediated neovascularization was elevated in **β3**-null/Tie1-Cre^+^ pSico-Con treated mice when compared with similarly treated wild-type/Tie1-Cre^+^ controls. pSico-Flk-1 treatment inhibited significantly VEGF-induced angiogenesis in both genotypes. However, pSico-Rac1 treatment had no effect in wild-type/Tie1-Cre^+^ mice, but did inhibit neovascularization in **β3**-null/Tie1-Cre^+^ mice when compared with pSico-Con controls (**Figure 6A**). Additionally, using a second approach, VEGF-induced angiogenesis in scrambled control-siRNA (Con siRNA) injected sponges in **β3**-null mice was elevated when compared with Con siRNA treated sponges in wild-type mice, and Rac1-specific-siRNA injection into sponges inhibited significantly angiogenic responses in **β3**-null, but not wild-type, mice **(** **Figure 6B** **).** Taken together these data suggest that Rac1 expression in endothelial cells is not essential for VEGF-mediated angiogenesis in wild-type mice but is involved in such responses in **β3**-null mice *in vivo*.

Further analysis of the role of Rac1 in VEGF-induced neovascularization responses was carried out using *ex vivo* aortic ring assays. Thoracic aortic rings from wild-type and **β3**-null mice were transfected with either a scrambled control (Con) or Rac1-specific siRNA. Rac1 mRNA and protein levels were reduced in aortic rings from both genotypes by approximately 85% after transfection with Rac1-specific siRNA (**Figure 6C**). These rings were embedded in Matrigel in the presence of VEGF alone or VEGF plus DC101, an antibody that blocks Flk-1 activity [27]. Quantitation of microvessel outgrowth showed that Rac1-depletion did not alter wild-type microvessel sprouting, but inhibited significantly the enhanced numbers of microvessel sprouts from **β3**-null aortas (**Figure 6D**). In addition, DC101 treatment significantly affected this process in Con- and Rac1-siRNA transfected aortic rings from both genotypes (P<0.01, **Figure 6D**).

### Rac1-depletion does not affect the levels or activity of other tested Rho GTPases in either wild-type or β3-null endothelial cells

**Figure 7:**
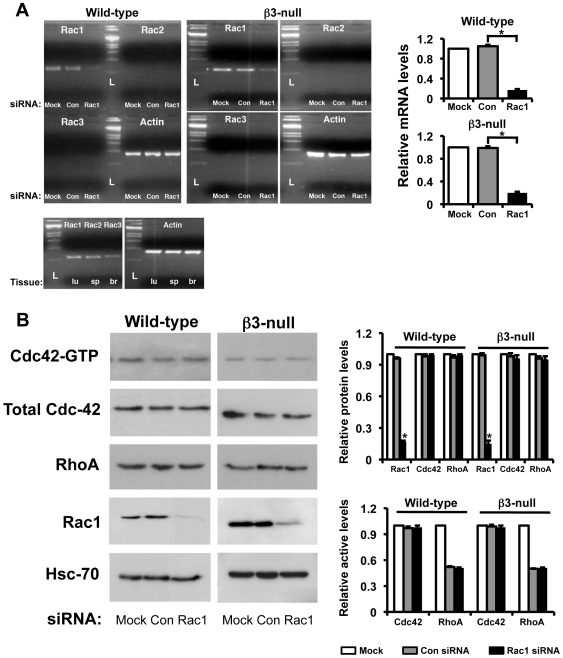
Rac1-depletion in endothelial cells does not affect the expression and activity of other Rho-related GTPases. **A.** Analysis of mRNA expression of Rac isoforms by RT-PCR. RNA was extracted from wild-type and β3-null endothelial cells transfected with Con or Rac1 siRNA. As expected, mRNA expression of Rac1 was significantly reduced in Rac1-depleted cells of both genotypes (*P<0.001). Rac2 and Rac3 isoforms were not detected in either Con- or Rac1-siRNA transfected endothelial cells. Extracts of lung (lu), spleen (sp) and brain (br) act as positive controls for Rac1, Rac2 and Rac3, respectively. Actin mRNA provided the internal control. L: ladder. Bar graphs represent relative mRNA levels of Rac1 after Mock- (white), Con- (grey) and Rac1-siRNA (black) treatment in both genotypes. **B.** Western blot analyses show that Rac1 expression, as expected, was significantly reduced in Rac1-depleted cells of both genotypes (*P<0.01). In contrast, Cdc42 and RhoA protein expression was not significantly affected in endothelial cells after Rac1-siRNA in both genotypes. In addition, for both wild-type and β3-null endothelial cells active levels of Rac1, Cdc42 and RhoA were examined using GST-PAK pull-down (Rac1 and Cdc42) and G-LISA® (RhoA) assays on Mock (white), Con- (grey) and Rac1-siRNA (black) transfected cells. Bars graphs show mean relative protein (top) and active (bottom) levels (+ s.e.m.) of Rac1, Cdc42, and RhoA in Mock (white), Con- (grey) and Rac1-siRNA (black) treated wild-type and β3-null cells. N = 3 independent experiments.

To begin to understand the mechanisms responsible for the biological effects of Rac1-depletion in wild-type and **β3**-null endothelial cells, we examined first the levels and activity of Rac2, Rac3, Cdc42 and Rho. Rac1-specific siRNA treatment of microvascular endothelial cells isolated from wild-type or β3-null mice reduced significantly Rac1 mRNA levels (**Figure 7A**), total protein levels (**Figure 7B**), and active Rac1 levels (**Figure 1A**). In contrast, transfection of endothelial cells using Con siRNA had no effect on Rac1 expression in either genotype. Importantly, no compensation by expression or activity of other tested Rho-related GTPases (Rac2, Rac3, Cdc42 and RhoA) were observed in either wild-type- or β3-null-Rac1-depleted endothelial cells (**Figure 7B**). These results suggest that the lack of an inhibitory response to Rac1 loss in wild-type endothelial cells is not due to compensation by either Rac2, Rac3, Cdc42 or RhoA. In addition, the dependency of **β3**-null endothelial cells on Rac1 is not due to any intrinsic change in these Rho GTPases.

### Rac1 is not essential for VEGF-mediated endothelial cell proliferation, scratch closure or tube formation in 2-dimensions

Given that Rac1 has been implicated in regulating positively cell migration, proliferation and tube formation, at least *in vitro*, we decided to examine also whether Rac1-depletion in **β3**-null endothelial cells affects these processes.

**Figure 8:**
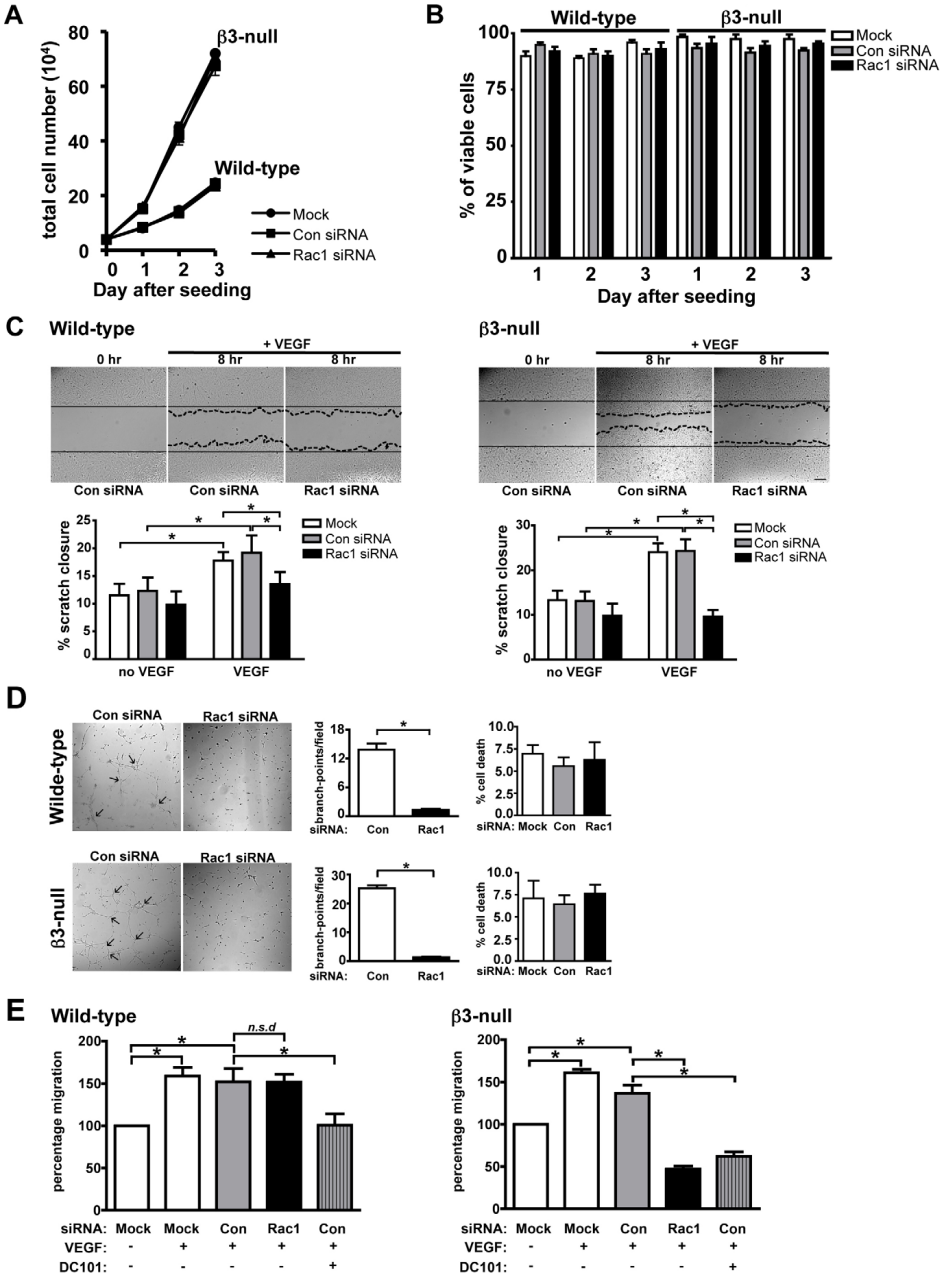
Effect of Rac1-depletion in wild-type and β3-null cells in 2D versus 3D. **A.** Endothelial cell growth over 3 days was assessed by counting the total number of Mock, Con- or Rac1-siRNA transfected wild-type and β3-null endothelial cells. Although β3-null cells grew faster than wild-type, Rac1-depletion did not affect cell growth in either genotype. Symbols (•, Mock; ▪, Con-; and ▴, Rac1-siRNA) values given represent mean total cell numbers (+ s.e.m) from 3 independent experiments. **B.** Trypan blue exclusion, used to assess cell viability, shows that Rac1 knockdown has no effect on endothelial cell viability in either genotype. Bar graph represents mean percentage of viable cells (+ s.e.m) from 3 independent experiments. **C.** Wild-type and β3-null endothelial cell migration in response to VEGF was assessed in scratch wound healing assays. Representative photomicrographs (20 x magnification) of scratches at 0 hr and after 8 hr of migration after VEGF (25 ng/ml) stimulation. Bar graphs represent the mean percentage migration relative to 0 hours (+ s.e.m.), from 3 independent experiments, displayed by Mock (white), Con- (grey) and Rac1-siRNA (black) transfected wild-type and β3-null endothelial cells. Under 2D conditions, Rac1-depletion inhibited VEGF-induced migration of both wild-type and β3-null endothelial cell cells to baseline levels. **D.** VEGF-stimulated endothelial cell tube formation was assessed in 2D Matrigel cultures. Representative phase contrast micrographs (20 x magnification) showing cord structures (arrows) that formed 8 hr after seeding of Con- or Rac1-siRNA transfected cells on 2D Matrigel. Bar graphs represent mean numbers of branch points per field (+ s.e.m.) from 3 independent experiments. Rac1-depletion inhibited significantly VEGF-mediated tube formation by both wild-type and β3-null endothelial cells (*P<0.0001). Trypan blue exclusion assays demonstrated that the percentage of cell death of cells isolated from the cord-formation experiment did not change. Bar graph represents mean percentage of cell death (+ s.e.m.) from 3 independent experiments. **E.** *In vitro* VEGF-mediated endothelial cell migration was assessed in a modified 3D Boyden chamber assay. Quantification of VEGF-mediated migration of Mock, Con- or Rac1-siRNA transfected wild-type (left) and β3-null (right) endothelial cells in the presence or the absence of DC101. Bar graphs represent mean percentage migration relative to Mock treated cells in the absence of VEGF (+ s.d). VEGF-mediated migration was significantly reduced in Con siRNA DC101 treated endothelial cells, confirming the requirement of Flk-1 in VEGF-induced migration and provides a control (*P<0.05) siRNA targeting of Rac1 did not affect wild-type (*n.s.d*), but did inhibit β3-null, endothelial cell migration in 3D conditions (*P<0.01). N = 3–5 independent experiments.

Analysis of endothelial cell growth by seeding equal numbers of Mock, Con- or Rac1-siRNA transfected wild-type or **β3**-null endothelial cells and counting the total number of cells per condition between 0 and 3 days showed no significant differences between Rac1-depleted and control cells in either genotype despite the increased growth rates of **β3**-null endothelial cells (**Figure 8A**). In addition, Trypan blue exclusion showed that the percentage of cells that were viable did not change after Rac1-depletion between 1-3 days after seeding in either genotype (**Figure 8B**). These data suggest that Rac1-knockdown does not inhibit the proliferation or viability of wild-type or **β3**-null endothelial cells in culture. In contrast, Rac1-depletion inhibited VEGF-mediated 2-dimensional (2D) scratch closure (**Figure 8C**) and cord formation (**Figure 8D**) when compared with Mock and Con siRNA controls without affecting cell viability (**Figure 8D**). These results confirm the reported role of Rac1 in promoting VEGF-mediated endothelial cell migration and cord formation conducted in standard 2D tissue culture conditions. However, given that the requirement for Rac1 in 3-dimensional (3D) versus 2D migration may be different [30], [31], and that our *in vivo* data did not show a requirement for Rac1 in wild-type angiogenesis, the effect of Rac1-depletion in endothelial cell migration was examined further in a 3D-modified Boyden chamber assay. Importantly, knockdown of Rac1 had no effect on VEGF-induced 3D chemotactic migration of wild-type endothelial cells, but did inhibit this mode of migration in **β3**-null endothelial cells. As controls wild-type endothelial cells transfected with Con siRNA and treated with DC101 showed baseline migration and this was further inhibited in similarly treated **β3**-null cells (P<0.05, **Figure 8E**). These results suggest that the role of Rac1 in 3D and 2D is different.

### Rac1 depletion does not affect VEGF-receptor2 expression

**Figure 9:**
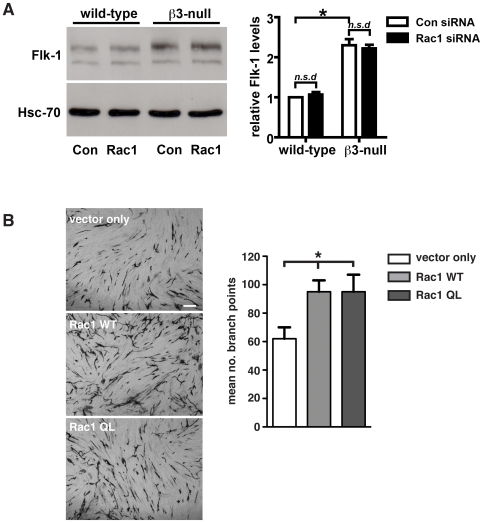
Endothelial Rac1-depletion does not affect the expression of Flk-1. **A.** Western blot analysis of Flk-1 expression levels in primary wild-type and β3-null endothelial cells transfected with scrambled (Con siRNA) or Rac1 (Rac1 siRNA) siRNAs. Although Flk-1 expression was increased significantly in β3-null endothelial cells when compared with wild-types (*P<0.01), Rac1-depletion did not affect Flk-1 levels (*n.s.d*) in either genotype. Bar graph represents mean (+ s.e.m.) of Flk-1 expression relative to Hsc-70 loading control (N = 3–4 independent experiments). **B.** HUVEC were transfected with vector only, a wild-type Rac1 construct (Rac1 WT), or a constitutively active Rac1 construct (Rac1 QL) and seeded on confluent fibroblasts. Tubules were visualized by PECAM1 staining 5 days after seeding. The mean number of branch points (+SEM) is shown in the accompanying bar chart. (n = 12 microscopic fields per condition). Scale bar: 100 µm.

Since we have shown previously that the loss of **β3**-integrin can elevate VEGF-mediated responses, VEGFR2/Flk-1 levels and Flk-1 activity [12], [27] we sought to determine the effect of Rac1-depletion on Flk-1 levels in wild-type and **β3**-null endothelial cells. Expression of Flk-1 was analyzed in primary wild-type and β3-null endothelial cells after transfection with either scrambled-control or Rac1-specific siRNA. Western blot analysis recapitulated our previous work showing that β3-null endothelial cells express higher levels of Flk-1 than wild-type controls. However, Rac1-depletion did not affect Flk-1 levels in either genotype (**Figure 9A**). This suggested that the elevated Rac1 activity that occurs in β3-null endothelial cells is downstream of changes in VEGFR2.

Although Rac1 appears dispensable to normal *in vivo* angiogenesis, the question remained as to whether elevated Rac1-activity could influence angiogenesis. To test whether elevated Rac1-activity influences 3D tube formation *in vitro*, both WT Rac1 and constitutively active Rac1 (Rac1 QL) were over-expressed in human umbilical endothelial cells (HUVEC) plated on a confluent layer of fibroblasts [32]. Tubules were visualized by PECAM-1 staining after 5 days in culture. The number of branch points in HUVEC overexpressing either form of Rac1 was significantly higher when compared to vector only transfected controls (*P*<0.02, **Figure 9B**), demonstrating that elevated Rac1 levels or activity can enhance tube formation.

Overall, our findings suggest that normally (in adult wild-type mice) angiogenesis does not depend on Rac1. However, when **β3**-integrin levels are altered, tumor growth, tumor angiogenesis and VEGF-driven angiogenesis are enhanced and dependent on Rac1.

## Discussion

Here we describe the role of Rac1 in tumor growth, tumor angiogenesis and VEGF-induced angiogenesis *in vivo*. Importantly, none of these processes were affected when Rac1 was depleted or deleted in the tumor endothelium of adult wild-type mice. These findings reveal that, unexpectedly, Rac1 expression in adult endothelial cells is not essential for tumor angiogenesis. However, we report that active levels of Rac1 are increased in β3-null endothelial cells and that the enhanced tumor growth, tumor angiogenesis and VEGF-mediated responses in β3-null mice were all dependent on endothelial Rac1 expression since Rac1-depletion in **β3**-null endothelial cells reduced significantly these responses. These data demonstrate that the requirement for endothelial-Rac1 becomes important when vascular β3-integrin is absent.

Endothelial cell proliferation, migration and tube formation are essential processes during tumor angiogenesis and Rac1 activity is believed to be important for these cellular processes *in vitro* implying a possible requirement for this molecule in angiogenesis *in vivo*. However our data demonstrating that tumor angiogenesis and VEGF-induced angiogenesis *in vivo* were not affected in either pSico-Rac1-infected wild-type/Tie1-Cre^+^ mice or tamoxifen-treated Rac1 flox/flox PDGFB-iCreER mice, imply that angiogenic processes such as proliferation, migration and tube formation *in vivo* are not affected by the induced-loss of Rac1 in adult endothelial cells. In contrast, recent studies have suggested a positive role for Rac1 in endothelial cell function. For example, a 50% loss of Rac1 in Tie2-Cre mice results in reduced neovascularization in the hind-limb ischemia model [24]. In addition, Rac1-deletion in Tie2-Cre embryos results in early lethality due to severe heart and vasculogenic defects by E8.5 [25]. The apparent discrepancies between our results and those reported in Tie2-Cre mice may be due to several reasons. For example, the haploinsufficiency or deletion of Rac1 in these systems is from early embryogenesis and this has been shown to induce changes in blood pressure and vasodilation [24] that, in turn, are known to affect neovascularization indirectly [33] and therefore it is likely that such changes in blood pressure would actually affect angiogenesis in the Rac1-haploinsufficient-Tie2-Cre mice. In contrast, using the pSico or PDGFB-iCreER-inducible systems we have deleted Rac1, predominantly, in adult tumor or VEGF-induced neo-endothelial cells themselves, thus reducing any side effects on the whole vasculature. In addition, Cre activity in Tie2-Cre mice, as well as being present in endothelial cells, is also apparent in approximately 80% of hematopoietic cells [34], [35], and it is tempting to speculate that the haploinsufficiency or deletion of Rac1 in this compartment may well affect angiogenesis. In contrast, in the pSico models that we have used, it is unlikely that the hematopoietic compartment is subject to Rac1-deletion and thus these cells are unlikely to affect angiogenesis in our system.

Regarding proliferation, our results corroborate the findings that increased Rac1 activity correlates with enhanced endothelial proliferation, since the elevated Rac1-activity in **β3**-null endothelial cells show elevated proliferative capacity [36]. However, our data go on to demonstrate that the loss of Rac1-activity is not sufficient to control microvascular-endothelial cell growth in culture and this is in line with other data where deletion of Rac1 does not affect cell proliferation [37].

Our data also illustrate that the requirement for Rac1 in 3D versus 2D endothelial cell migration is different. Indeed, by examining VEGF-induced endothelial cell migration in 2D (scratch wound healing assays) and in 3D (3D modified Boyden chamber assays), Rac1-depleted cells were less motile than controls in 2D; whereas they migrated as well as controls in 3D. Several studies have shown that VEGF-mediated endothelial cell migration in 2D involves activation of Rac1 [38], [39], [40] and our data are in line with these observations. Importantly, however, our findings demonstrate that the involvement of Rac1 in 3D endothelial cell migration is not as critical as originally thought and this is likely to reflect the reason for the normal tumor angiogenesis in Rac1-depleted mice *in vivo*.

The reduced VEGF-induced 2D tube formation, after Rac1-depletion in endothelial cells, that we observed is also in line with several reports [41], [42], [43], [44], [45], [46], [47]. In contrast, using a novel 3D model involving co-culture with VEGF-A-overexpresing fibroblasts, others have shown that dominant-negative N17Rac1 has no effect on VEGF-induced capillary-like network formation of endothelial cells [48]. These investigators showed that a dominant negative mutant of N-Ras (a small GTPase from a different family to that of Rac1) suppressed capillary-network formation of endothelial cells induced by VEGF-A-producing fibroblasts whereas the mutants of Rac1, RhoA or Cdc42 did not. Our observations also support the notion that, in 3D environments, Rac1 is dispensable for VEGF-mediated capillary-formation in wild-type but not **β3**-null aortic rings and angiogenesis *in vivo*.

Previous studies have suggested that β3-integrin activation induces Rac1 activity. For example, inhibition of endothelial cell cyclooxygenase-2, by nonsteroidal anti-inflammatory drugs, suppresses αvβ3-dependent activation of Rac1 [49], [50]. Given such crosstalk between integrins and Rac1, it is surprising that Rac1-activity is enhanced in β3-null endothelial cells. The increased active levels of Rac1 may reflect a compensatory response due to the deletion of β3-integrin, since rescuing the expression of β3-integrin in β3-null endothelial cells diminished the activity of Rac1 to levels similar as those seen in wild-type. Importantly, however, the expression and activity levels of other tested Rho-related GTPases (e.g. Rac2, Rac3, Cdc42 and RhoA) were equal in wild-type and β3-null endothelial cells suggesting that these Rho GTPases are unlikely to compensate for the loss of **β3**-integrin.

Although recent work has shown that molecules such as RhoG can compensate for Rac-1 [51] there is also evidence that this depends on specific signaling cascades and is not always the case [52]. Our data suggest that this is unlikely in β3-null endothelial cells, since we have shown, that by inhibiting Rac1 expression alone, we can reduce the enhanced angiogenic responses of β3-null mice back to levels observed in wild-type mice either with or without depletion of Rac1. Although it is tempting to speculate that RhoG activity may compensate for Rac1 in wild-type endothelial cells, thus explaining the reduced requirement for Rac1 in wild-type angiogenic events *in vivo*, this is also not likely because Rac1-deletion in both β3-null and wildtypes brings tumor growth and blood vessel density to the same level in both genotypes. Thus the most logical assumption is simply that Rac1 is not involved in tumor angioegenesis *in vivo* unless β3-integrin is absent.

Our data indicate that the importance of Rac1 in angiogenic processes is revealed only after **β3**-integrin is absent. We show that the enhanced Rac1 activity in β3-null endothelial cells is responsible, at least in part, for the elevated tumor angiogenesis and VEGF-mediated angiogenic responses in β3-null mice.

Despite the fact that β3-null endothelial cells have elevated VEGFR2 (Flk-1) levels [12], [27], [53], even after Rac1-depletion, indicates that elevated VEGFR2 levels alone are not sufficient to enhance angiogenesis. We show that VEGF/VEGFR2-mediated responses are Rac1-dependent in the absence, but not in the presence of β3-integrin, raising the possibility that downstream signalling of VEGFR2 can either involve Rac1 or not, but that this depends on β3-integrin.

In **β3**-null mice the increased Rac1-1 activity likely results in changes in downstream signaling that confer the enhanced angiogenic responses. For example, p38 MAP kinase signaling is known to be downstream of Rac1 and **β3**-integrin and plays a role in angiogenic processes such as endothelial cell migration and regulation of cell-cell adhesion [54], [55], [56]. Thus, in future experiments it would be of interest to examine the role of p38 MAP-kinase signaling in **β3**-null endothelial cells.

Several studies have shown that the blockade of αvβ3 using monoclonal antibodies or antagonist peptides can inhibit tumor growth and tumor angiogenesis in mouse xenografts models and other preclinical tests [57] and some of these agents, such as the humanized monoclonal antibody Vitaxin [5] and the cyclic RGD peptide Cilengitide [3], [4], [6], [7] are in clinical trials for the anti-angiogenic treatment of cancer. However, to date, there is very little evidence showing these inhibitors can in fact suppress tumor angiogenesis in humans [3], [4], [6], [9], [10], [11]. This may simply reflect that tumor angiogenesis is not actually dependent on the activity of αv**β3**-integrin in endothelial cells in several tumor types. Indeed, in some human tumors, such as angiosarcomas, endothelial β3-integrin expression levels decrease as malignant transformation progresses [16]. In addition, vascular β3-integrin levels were significantly lower in lung metastases from colorectal carcinomas when compared with those seen in primary colorectal tumors [17], [18]. Thus, targeting β3-integrin might not be a useful anti-angiogenic strategy for this particular subset of patients.

One question that remains unanswered is: “when is β3-integrin actually bound to ligand during the angiogenic process?” Wow-1 is an antibody that recognizes ligated β3 integrin in cells in culture [58] but, unfortunately, has yet to be proven to work in whole tissue sections or *in vivo*. Thus testing β3-integrin ligation *in vivo* is presently impossible without the use of inhibitors. Importantly, since inhibition of β3-ligation does not phenocopy the deficiency of β3-integrin, where such angiogenic responses are enhanced [12], it is also not possible to test whether β3-integrin ligation is truly relevant to our *in vitro* findings. This is because inhibition of β3-ligation, using RGD mimetics or antibodies, can inhibit tubule formation, for many reasons including an increase in cell death [59]. Thus inhibiting β3-integrin function not only inhibits β3-ligation but also simultaneously alters downstream signaling. Furthermore, we have recently shown that low doses of such agents can actually enhance angiogenesis, both in vitro and in vivo, but that these doses do not affect ligand binding [60]. Thus in conclusion, although such experiments would be of great interest we await the technology to enable us to test the effect of β3-ligation effectively *in vivo* without the use of inhibitors or knockout mice.

Our findings suggest that inhibition of Rac1 might be an effective anti-angiogenic therapeutic strategy in tumor blood vessels that display low levels of β3-integrin. Furthermore, combination therapy of anti-Rac1 and anti-β3-integrin targeted to endothelial cells may be an effective general anti-angiogenic strategy.

## Materials and Methods

### Ethics Statement

All procedures on mice were performed in accordance with the United Kingdom Home Office regulations.

### Mice

β3-integrin-deficient and wild-type Tie1-Cre mice used for analysis of pSico lentiviruses *in vivo* were generated by crossing β3-integrin-deficient [61] and wild-type mice with Tie1-Cre transgenic mice ([62]; kindly provided by Prof. Fässler, Max Plank Institute of Biochemistry, Martinsried, Germany), both on a mixed C57BL6/129 background. β3-integrin-deficient and wild-type mice were also bred with the Immortomouse® (Charles River Laboratories, MA, USA) on a C57BL6 background and were used for preparation of immortalised endothelial cell cultures employed in some of the *in vitro* assays. Inducible vascular endothelium-specific Rac1-deficient mice (Rac1 flox/flox PDGFB-iCreER) were generated by crossing Rac1 flox/flox mice on a mixed C57BL6/129 background [28] with PDGFB-iCreER transgenic mice on a mixed C57BL6/CBAF2 background [29].

### Isolation of mouse endothelial cells

Mouse lung endothelial cells (MLEC) were isolated as previously reported [63]. Briefly, minced lungs were digested in collagenase type 1 (Gibco) and passed trough a 70 µm pore size cell strainer (BD Falcon) and cell suspension plated onto tissue culture plates coated with 0.1% gelatin containing 10 µg/ml fibronectin (Sigma) and 30 µg/ml Purecol (Nutacon). When cells reached ∼80% confluency, macrophages were removed from the culture by immunosorting using rat anti-mouse CD16/CD32 Fcγ receptor antibodies (AbD Serotech) and with two subsequent sorts using rat anti-mouse ICAM-2 antibodies (AbD Serotech) endothelial cells were positively selected. Endothelial cell purity was 96–98% as assessed by performing flow cytometry for PECAM-1 (clone MEC 13.3, Pharmingen), Endoglin (Pharmingen) and ICAM-2 expression. Acquisition of samples was performed on a FACS Calibur fluorescence activated cell sorter (BD Biosciences) and the data were analysed with CELLQUEST software (BD Biosciences). Cells were cultured routinely in a 50∶50 mix of Hams F12 and D-MEM media supplemented with 20% FCS, 6 mM glutamine, 25 µg/ml endothelial mitogen (Biogenesis), 1 µg/ml heparin and antibiotics.

### Generation of pSico-Rac1 lentivirus

The plasmid for Stable RNA interference conditional (pSico) was a kind gift from Dr. Tyler Jacks (Massachusetts Institute of Technology, MA, USA) [64]. The pSico-Rac1 lentiviral vector was produced by digesting the pSico construct with HpaI-XhoI restriction enzymes (Promega), inserting shRNA oligonucleotides to target the mouse Rac1 gene and religating using T4 DNA ligase (Promega). A pSico containing shRNA to target the firefly Luciferase gene (pSico-Con) was generated to serve as a non-specific control RNAi and a pSico containing shRNA to target the mouse VEGFR2/Flk-1 gene (pSico-Flk-1) was made to stably knockdown the expression of Flk-1, therefore serving as a positive control in angiogenesis assays. Oligonucleotides coding shRNA for mouse Rac1, mouse VEGFR2/Flk-1, *β*3-integrin [65] and firefly Luciferase were designed using the program PSICOLIGOMAKER 1.5 (available on Jacks' website). Luciferase oligos were blasted against the mouse genome to ensure that they do not recognise any sequence in the mouse DNA. All oligonucleotide sequences are available upon request. Chemically competent E. coli (One Shot Stbl3, Invitrogen) were transformed with ligated DNA. Plasmids were purified (QIAprep kit, Quiagen), digested with SacII-NotI (Promega) and positive clones released a fragment approximately 50 bp larger than the fragment released by pSico empty vector. Positive clones were sequence verified (Equipment Park Laboratory, London Research Institute Cancer Research UK). Sequences were analysed using DNA Strider 1.4f1 software (CEA, France).

Lentiviruses were generated essentially as described in the Virapower Lentiviral Expression System User Manual (Invitrogen). Briefly, 3 µg of pSico-Rac1, pSico-Con or pSico-Flk-1 lentiviral vectors and 9 µg of ViraPower Packaging Mix (Invitrogen) were cotransfected in 293FT cells (Invitrogen) by using Lipofectamine 2000 reagent (Invitrogen). Supernatants were collected 48–72 hr after transfection, centrifuged at 3000 rpm for 15 min, filtered through a 0.45 µm filter, and used to directly infect primary mouse lung endothelial cells. Overnight infection usually was sufficient to infect 50–65% of the cells as assessed by flow cytometry. Acquisition of samples was performed on a FACS Calibur fluorescence activated cell sorter (BD Biosciences) and the data were analysed with CELLQUEST software (BD Biosciences). GFP-positive endothelial cells were sorted 3–4 days after transfection using a FACSAria cell sorter and program. Sorted cells were used to assess efficiency of Cre-mediated RNAi in vitro by transfecting them with Cre expression-plasmid, pTURBO-Cre (a kind gift from Dr. David Stevenson, The Beatson Institute, UK). Transfection of cells was performed by electroporation using the Basic Nucleofector kit designed for primary mammalian endothelial cells (Amaxa GmbH) following the manufacturer's instructions. One week after transfection with pTURBO-Cre, Rac1 and Flk-1 knockdown efficiencies were assessed by RT-PCR and Western blotting. For in vivo experiments, lentivirus were ultracentrifuged at 25000x g for 1.5 hr and stocks titrated. Titers ranged between 1.5–2×10^6^ i.u/ml.

### Rac1 siRNA

To target mouse Rac1 gene we used siGENOME ON-TARGETplus SMARTpool reagent (Dharmacon). Primary and immorto MLEC and mouse aortic tissue (see below) were transfected with anti-mouse Rac1 siRNA using Oligofectamine reagent (Invitrogen) following the manufacturer's instructions. The final concentration of oligonucleotides in the transfection mixture was 100 nM and gene expression was examined 48 hr (MLEC) or 5 days (aortic tissue) post-transfection by RT-PCR and Western Blot analysis. In all transfection experiments scrambled siRNA (Dharmacon)-transfected samples were used as controls.

### Semiquantitative RT-PCR

We used Rac1 isoform-specific primers derived from cDNA mouse sequences and PCR conditions that have been described previously [37]. As controls of the Rac2 and Rac3 reactions, total RNA and subsequent cDNA synthesis from spleen and brain tissues were obtained. Actin RT-PCR was performed as a loading control.

### Western blot analysis

Cells were grown to ∼80% confluency and lysed with RIPA buffer (50 mM Tris-HCl pH 7.4, 1% NP-40, 0.25% sodium deoxycholate, 150 mM NaCl, 1 mM EDTA, 1 mM sodium orthovanadate, 1 mM NaF, 1 mM PMSF), supplemented with protease inhibitor cocktail II (Calbiochem). For preparation of lysates from aortas, 12–15 aortic rings were homogenised in 50 ml RIPA buffer by using a pestle and microwaved for 10–15 sec. Homogenates were centrifuged at 10,000 rpm for 10 min at 4°C, to pellet insoluble material. Lysates were subjected to SDS-PAGE and transferred to membranes (Hybond-ECL, Amersham Biosciences). Membranes were blocked in either 5% milk or 5% BSA in PBS-Tween (0.05%) and incubated with mouse anti-Rac1 (Clone 23A8, Upstate), mouse anti-Cdc42 (Chemicon), mouse anti-RhoA (Santa Cruz) or rabbit anti-VEGFR2 (Cell Signalling Technologies) antibodies. Membranes were washed three times in PBS-Tween (0.05%) and subsequently incubated with HRP-conjugated IgGs. Immunoreactive bands were visualised by incubating the membranes with ECL chemiluminescence reagents (Amersham Life Science) and exposing the membranes to autoradiography film (Amersham Life Science). After detection of the protein of interest, membranes were stripped with stripping solution (Chemicon), and re-blotted with mouse anti-Hsc-70 antibody. Densitometry was performed using a gel acquisition and analysis set-up (UV Products, Ltd.). Band densities were normalised to Hsc-70 to make quantitative measurements of the protein of interest.

### Rho GTPases activity assays

A construct encoding glutathione S-transferase (GST) linked to the amino acid residues 57 to 141 of the PAK-CRIB domain (GST-PAK-CRIB; kindly provided by Dr. John Collard, King's College, London, UK and Dr. Vania Braga, Imperial College, London, UK), was used to pull down active Rac1 and Cdc42 from MLEC cell lysates transfected with scrambled or Rac1 siRNA. Pull-downs were performed as described [66]. Samples of lysate preincubation (total protein) and postincubation with the GST-PAK-CRIB beads (active protein) were resolved in 12% polyacrylamide gels, transferred to PVDF membranes, and immunoblotted with anti-Rac1 or anti-Cdc42 antibodies as described above. The proportion of active Rac1 and Cdc42 relative to their the total amount in the cells was determined by densitometry (UV Products, Ltd.). Active levels of RhoA in MLEC transfected with scrambled or Rac1 siRNA, were assessed using the G-LISA^TM^ RhoA activation assay Biochem Kit^TM^ (Cystoskeleton) following the manufacturer's instructions. The degree of RhoA activation was determined by comparing absorbance readings at 490 nm from Rho control protein (constitutively active RhoA protein) samples versus cell lysates of interest.

### In vivo tumorigenesis and endothelial-specific Rac1-deletion

Rac1 flox/flox and Rac1 flox/flox PDGFB-iCreER mice were implanted subcutaneously with tamoxifen (Rac1 flox/flox and Rac1 flox/flox PDGFB-iCreER) or placebo (Rac1 flox/flox PDGFB-iCreER) 1 mg-slow release pellets in the scruff of the neck. One day later B16F0 tumor cells (1×10^6^) were injected subcutaneously in the flank. At day 10 after tumor cell injections, tumors were dissected out and processed for histology.

### In vivo VEGF-induced angiogenesis assays

Mouse subcutaneous sponge angiogenesis assays [67] were carried out by implanting two autoclaved sponges subcutaneously into the flanks of wild-type/Tie1-Cre+ and β3-null/Tie1-Cre+ mice. Sponges were injected in situ as follows: on days 1, 5 and 9 with 100 µl of 10 ng/ml VEGF-A164; on days 3, 7 and 13 with pSico-Con, pSico-Rac1 and pSico-Flk-1 (10^6^ i.u/ml) in 100 µl of 10 ng/ml VEGF-A164. At day 15 after sponge implantations, the mice were euthanized by CO_2_ asphyxiation, sponges excised and fixed for paraffin embedding. Additionally growth factor induced angiogenesis assays were carried out by implanting two autoclaved sponges subcutaneously into the flanks of wild-type and β3-null mice. These sponges were further injected in situ every 2 days with Con siRNA (3 mg) in 100 µl of PBS alone, Con siRNA (3 mg) or Rac1 specific-siRNA (3 mg) in 100 µl of PBS containing 10 ng/ml VEGF-A164. At day 15 after sponge implantation, the mice were euthanized by CO_2_ asphyxiation, sponges excised and processed for histology.

### Quantification of blood vessel density

Tumor and sponge samples were snap frozen for cryosectioning or fixed in 4% paraformaldehyde for paraffin-embedding, respectively. Frozen sections were brought to room temperature, fixed in 4% PFA and rehydrated in PBS, whilst paraffin embedded sections were deparaffinized, rehydrated in PBS and microwaved on full power (950 W) in sodium citrate buffer pH 6.0, before incubation with rat anti-mouse PECAM-1 (Pharmingen), rabbit anti-green fluorescent protein (GFP) (Mol Probes) and rat anti-mouse endomucin (kindly provided by Dr. Dietmar Vestweber, Max-Planck-Institute of Molecular Biomedicine, Muenster, Germany) followed by incubation with goat anti-rat Alexa Fluor-546, goat anti-rabbit Alexa Fluor-488 (both from Mol Probes) and biotinylated rabbit anti-rat antibody (Vector Laboratories), respectively. When biotinylated antibody was used an alkaline phosphatase-based system (VECTASTAIN ABC-AP, Vector Laboratories, Peterborough, UK) with Vector Blue substrate kit (Vector Laboratories, Peterborough, UK) was used to amplify the signal according to the manufacturer's instructions. Blood vessel density in immunofluorescent stained sections from size-matched tumors was determined by counting the number of PECAM-1^+^/GFP^−^ or PECAM-1^+^ blood vessels present across an entire section and then dividing by the total area of the section. Blood vessel density in immuno alkaline-phosphatase stained sections from sponges was determined by counting the number of endomucin+/GFP− stained blood vessels present across an entire section and then dividing by the total area of the section. In all cases blood vessel counting was performed in a double-blind fashion using an epifluorescence microscope Zeiss (Carl Zeiss, Jena, Germany) with associated digital camera (Hamamatsu Photonics, Ltd., Welwyn Garden City, UK) and using Open Lab software v4.1software (Improvission, Coventry, UK) to capture images and calculate areas of the sections.

### Ex vivo mouse aortic ring assays

Wild-type and β3-integrin-deficient mice were sacrificed by cervical dislocation, thoracic aortas removed under sterile conditions, periaortic fibroadipose tissue was removed and aortas were cut into 0.5–1 mm-wide rings. After 24 hours siRNA transfected aortic rings were rinsed in D-MEM supplemented with 6 mM L-glutamine and 2% FCS (basal medium) and each ring was transferred to a well of a 96-well plate containing polymerized growth factor reduced (GFR)-Matrigel (Becton Dickson, Beds, UK) before adding 150 µl of basal medium. The medium was supplemented with 25 ng/ml VEGF-A164 and DC101 at 20 µg/ml [27]. Where DC101 was used, the GFR-Matrigel was supplemented with 20 µg/ml DC101 antibody just before polymerisation. The cultures were grown at 37°C and 8% CO_2_, for up to 8 days. Aortic rings were examined microscopically and media including VEGF and DC101 was changed every 2 days. On day 5, phase contrast photomicrographs were taken using an inverted microscope (Carl Zeiss) equipped with a camera (Hamamatsu Photonics, Ltd.) and angiogenesis calculated by counting the number of capillary sprouts per ring. Rac1 expression in transfected aortic rings was assessed by using both semiquantitative RT-PCR and Western blot analysis.

### HUVEC Tubule Formation Assay

These assays were performed as described in [68]. The constructs used are described in [32].

### Mouse lung endothelial cell growth

Temperature-sensitive immortalised wild-type and β3-null mouse lung endothelial cells were plated at density of 1,500 per square centimetre on pre-coated tissue culture plastic. siRNA transfections were performed and cells were allowed to continue growing. Cells were trypsinized from triplicate wells every day for up to three days and counted using a haemocytometer (Fast Read 102 cell counting chamber [ISL, Paignton, UK]). Cell viability was assayed by Trypan blue exclusion method.

### Wound closure scratch assays

Wound closure scratch assays were performed as described (21). Briefly, immortalised β3-null and wild-type MLEC were plated at a density of 10,000 per square centimetre on pre-coated tissue culture plastic. siRNA transfections were performed and cells were grown until a confluent monolayer was achieved. Cells were serum starved for 3 hr, monolayers scratched with a P200 tip, and the scratches were photographed using an inverted microscope (Carl Zeiss Axioplan) equipped with a camera (Hamamatsu Photonics, Ltd.) at three points along their length. The medium was changed for 50∶50 mix DMEM:Hams F12 plus glutamine supplemented with VEGF-A164 (25 ng/ml) and cells allowed to migrate over 8 hr at 37°C. Scratches were rephotographed, and the percentage of migration was determined relative to the scratch width at 0 hr.

### Modified Boyden chamber assays: 3D migration assay

One day before setting up the 3D migration assays, Con- and Rac1- siRNA transfected β3-null and wild-type and immortalised MLEC were treated with 4 µg/ml Mitomycin C (Sigma, Dorset, UK) at 37°C for 2 hr, to avoid changes in cell proliferation. Cells were washed twice in PBS and serum starved overnight in D-MEM:Ham's F12 plus L-glutamine. Matrix solution was prepared by mixing 1 part of GFR-Matrigel with 1 part of D-MEM:Ham's F12 media. 70 µl of matrix solution was pipetted into the upper well of the transwell (Costar, MA, USA) and incubated at 37°C for at least 60 min. D-MEM:Ham's F12 plus L-glutamine and 2.5% FCS supplemented with growth factor alone or growth factor and inhibitor (VEGF-A164 [25 ng/ml] and DC101 [50 ng/ml]) were pipetted into the ‘lower chamber’. Cells resuspended in D-MEM:Ham's F12 plus L-glutamine and 2.5% FCS at 50000–75000 per transwell were seeded onto the ‘top chamber’ and allowed to migrate at 37°C, 8% CO_2_ for 72 hr. Analysis of migrating cells was carried out by counting the number of cells that passed through the transwell after that period. In each experiment, each sample/condition was always assessed in quadruplicate.

### Endothelial tube-like cord formation

Wild-type and β3-null immortalised Mock, Con- and Rac1-siRNA transfected MLEC were starved overnight in 1% FCS in DMEM:Hams F12 plus glutamine. GFR-Matrigel was diluted 1∶1 in DMEM:Hams F12 plus glutamine on ice, mixed or not with VEGF-A164 at 25 ng/ml final concentration, pipetted onto 10 mm glass coverslips and incubated at 37°C for 30 min. Cells at 120,000 cells/ml in DMEM:Hams F12 plus glutamine were plated per well/coverslip and incubated at 37°C. Every two hours cells were observed under an inverted microscope (Carl Zeiss Axioplan) equipped with a camera (Hamamatsu Photonics, Ltd.), and photographed. Branch points of developing tubular structures were counted and compared at 8 hr time-point.

### Analysis of Statistical Significance

Data sets were analysed for significance using Student's t test. P<0.05 was considered statistically significant. Experiments were carried out in a double blind fashion and all experiments were performed in triplicate.
